# Consequences of Early Separation of Maternal-Newborn Dyad in Neonates Born to SARS-CoV-2 Positive Mothers: An Observational Study

**DOI:** 10.3390/ijerph18115899

**Published:** 2021-05-31

**Authors:** Maria Giulia Conti, Fabio Natale, Ilaria Stolfi, Roberto Pedicino, Giovanni Boscarino, Camilla Ajassa, Viviana Cardilli, Giovanni Luca Ciambra, Laura Guadalupi, Paola Favata, Paola Repole, Francesca De Luca, Giulia Zacco, Roberto Brunelli, Gianluca Terrin

**Affiliations:** 1 Department of Maternal and Child Health, Policlinico Umberto I Hospital, Sapienza University of Rome, 00161 Roma, Italy; mariagiulia.conti@uniroma1.it (M.G.C.); fab.natale@libero.it (F.N.); ilaria.stolfi@gmail.com (I.S.); rpedicino@libero.it (R.P.); giovanni.boscarino@yahoo.com (G.B.); camilla.ajassa@uniroma1.it (C.A.); viviana.cardilli@uniroma1.it (V.C.); gianlu.ciambra@gmail.com (G.L.C.); lallagua@yahoo.it (L.G.); favata.paola@alice.it (P.F.); paolarepole@gmail.com (P.R.); francesca27deluca@gmail.com (F.D.L.); giuliazacco95@gmail.com (G.Z.); roberto.brunelli@uniroma1.it (R.B.); 2 Department of Molecular Medicine, Sapienza University of Rome, 00185 Roma, Italy

**Keywords:** breastfeeding, COVID-19, neonatal infection, neonatology

## Abstract

As the severe acute respiratory syndrome coronavirus 2 (SARS-CoV-2) infection continues its spread all over the world, data on perinatal management of the maternal-infant dyad are urgent. We performed an observational study to describe the effects of the early separation of the maternal-infant dyad, in case of maternal SARS-CoV-2 infection. We reported the medical records for 37 neonates born to 37 SARS-CoV-2 positive mothers in a setting of separation of the dyad after birth. Data on neonatal infection, clinical condition, and breastfeeding rate were recorded until the first month of life. No maternal deaths were recorded; 37.8% of women had at least one pregnancy-related complication. We reported a high adherence to recommended safety measures after discharged with 84.8% of the mothers using at least one personal protective device and 51.5% using all the protective devices. We reported one case of vertical transmission and no cases of horizontal transmission. However, the separation of the dyad had a negative impact on breastfeeding because only 23.5% of the newborns received exclusively human milk during the first month of life. Despite early separation of the dyad protecting the newborns from possible horizontal transmission of SARS-CoV-2, it negatively affects breastfeeding during the first months of life.

## 1. Introduction

Since the first Chinese-reported case in December 2019, the novel coronavirus disease (COVID-19), caused by severe acute respiratory syndrome coronavirus 2 (SARS-CoV-2), has been rapidly spreading out all over the world and was recognized as a pandemic by the World Health Organization (WHO) on 11 March 2020 [1]. In most European countries, the trend of SARS-CoV-2 infection showed a characteristic pattern, with two waves of rapid spread (a first wave from March to May and the second from September 2020) interrupted by a mild remission during summer. While the first wave of COVID-19 infection mainly affected northern Italy, the second wave appears more diffuse and is currently undermining the entire national health system and economy.

The new coronavirus, SARS-CoV-2, is genetically similar to SARS-CoV but is more contagious. Thus, it is crucial to limit potential person-to-person transmission by using all the necessary precautions. The SARS-CoV-2 might be transmitted from the infected mother to her newborn both vertically [2] and horizontally [3,4,5]. The different modalities of mother-to-child transmission were clearly defined and categorized in a recent report issued by the WHO [6]. However, the putative contribution of different factors involved in the horizontal transmission—including the separation of the dyad after delivery, breastfeeding, regular use of personal protective devices and housing environment—is still unclear.

The clinical guidelines released by the different societies of obstetrics and neonatology for the management of the maternal-infant dyad in case of confirmed maternal SARS-CoV-2 infection [7,8,9,10] acknowledged both the increased risk of horizontal transmission as a result of an early mother–child contact and the potential harmful consequences of an early forced separation (including the lack of skin-to-skin care practice and the protective properties of breastfeeding). It was recently suggested that rooming-in and breastfeeding can be practiced by infected women in stable clinical conditions and in a specific setting [11] including an adequate number of health care providers, dedicated spaces and appropriate equipment. This setting is not always feasible, so several birth centers were forced to separate SARS-CoV-2-infected mothers from their newborns soon after birth [12].

We present the results of a study on key neonatal outcomes in a setting of separation of the dyad soon after birth, in case of confirmed maternal SARS-CoV-2 infection.

## 2. Materials and Methods

### 2.1. Study Design and Population

We designed an observational study on SARS-CoV-2-positive mothers and their newborns, who were consecutively admitted at Policlinico Umberto I Hospital, Sapienza University of Rome, from 1 April 2020 to 18 March 2021. During the study period, Policlinico Umberto I Hospital was a reference center for pregnant women positive for SARS-CoV-2 in Rome, Italy. Maternal SARS-CoV-2 infection was confirmed upon hospital admission by a real-time reverse transcriptase-polymerase chain reaction (RC-PCR) on a nasopharyngeal swab sample. All newborns delivered by infected mothers were similarly tested for SARS-CoV-2, immediately after birth and cleaning, by nasopharyngeal swab with RT-PCR. Nasopharyngeal swab samples were also obtained from all neonates at day of life (DOL) 5 and DOL 10. This study followed the Strengthening the Reporting of Observational Studies in Epidemiology (STROBE) reporting guideline [13]. The study protocol was conducted in conformity with the World Medical Association Declaration of Helsinki for medical research involving human subjects and was specifically approved by the Ethical Committee of the Policlinico Umberto I Hospital. A written informed consent from all parents was obtained at the enrollment.

### 2.2. Management of the Mother-Infant Dyad

All the infected mothers were separated from their newborns immediately after birth and during the entire hospitalization, due to logistic reasons. At discharge, the dyad was recomposed, and breastfeeding was encouraged by medical staff after appropriate counseling on the safety conditions—handwashing and use of surgical mask and gloves—for the care of the baby.

### 2.3. Data Collection

We recorded clinical and demographic data of the mothers, including age, ethnicity, smoking habits, educational level, blood type, comorbidity conditions, premature birth, administration of antenatal steroids or intrapartum antibiotics, intrauterine growth restriction (IUGR) and type of delivery. We also collected data regarding SARS-CoV-2 infection, including clinical symptoms, hospital admission for COVID-19, radiologic diagnosis of SARS-CoV-2 related pneumonia and need of oxygen therapy. Neonatal clinical baseline characteristics, including gestational age (GA), birth weight (BW), gender, the presence of fetal distress, pH on cord blood, Apgar score at 5 min of life, days of weight gain, blood type, comorbidity condition, information about testing or potential symptoms of SARS-CoV-2 infection and length of hospital stay were collected.

At DOL 28, we checked for neonatal clinical conditions, and we collected data about feeding methods, use of mask and hygienic measures by the mother during baby care, characteristics of the habitation, number and health status (including their possible infection by SARS-CoV-2) of cohabitants who have been in contact with the neonate.

### 2.4. Statistical Analysis

The impact of maternal symptoms on obstetric and neonatal outcomes was assessed in the two subpopulations of the asymptomatic and symptomatic mothers. Statistical analysis was performed using the IBM Statistical Package for Social Science software (SPSS Inc., version 25.0 Chicago, IL, USA). We checked for normality using a Shapiro–Wilk test. The median and minimum-maximum range summarized continuous variables. Qualitative variables were expressed as number and percentage. We used an χ² test or exact test for categorical variable and *t*-test, Mann–Whitney and Wilcoxon tests for paired and unpaired variables. The level of significance for all statistical tests was two sided (*p* < 0.05).

## 3. Results

### 3.1. Obstetric and Clinical Features of SARS-CoV-2 Infected Mothers

We recorded 37 mothers with confirmed infection by SARS-CoV-2 and collected data on 33 maternal-infant dyads (four dyads were lost during follow-up). Maternal demographic characteristics were similar between asymptomatic and symptomatic mothers (Table 1). Symptomatic mothers had a higher educational level (Table 1). The trend observed in symptomatic patients towards an increased incidence of at least one pregnancy-related complication did not reach the threshold of statistical significance (Table 1).

Maternal symptoms for COVID-19 are listed in Table 2. No maternal deaths were recorded. Of the 37 mothers, 4 (10.8%) were transferred to a referral unit for COVID-19 in our hospital, 8 (21.6%) received a radiologic diagnosis of SARS-CoV-2 interstitial pneumonia, 3 (8.1%) required oxygen supplementation and 1 (2.7%) was transferred to ICU and treated with continuous positive airway pressure (CPAP).

### 3.2. Neonatal Outcomes

The characteristics recorded at birth were similar in the two groups of newborns delivered by symptomatic and asymptomatic mothers (Table 3).

**Figure 1:**
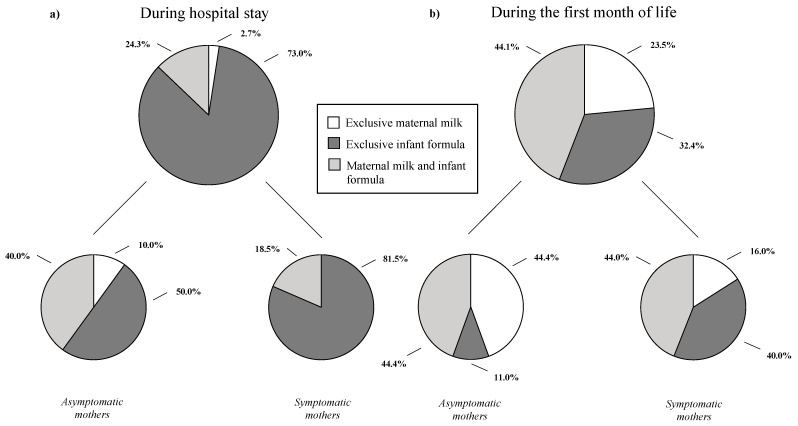
Feeding methods of the neonates enrolled in the study. As shown in the figure, (**a**) is “during hospital stay” and (**b**) is during the first month of life.

Neonatal comorbidities were exclusively recorded in the newborns from symptomatic mothers with an overall rate of 10.8%, including three neonates (8.1%) diagnosed with neonatal hemolytic disease and one neonate (2.7%) who presented mild respiratory distress at birth, respectively. We recorded one possible neonatal vertical infection (2.7%) defined by the detection of SARS-CoV-2 in nasopharyngeal swab obtained immediately after birth tested with RT-PCR and confirmed by a series of positive nasopharyngeal swabs collected at 3, 7, and 10 DOL. The newborn was isolated, admitted to the NICU for clinical surveillance and finally discharged at DOL 12. At discharge, one of the enrolled neonates (2.7%) received exclusively maternal milk, while most were fed with infant formula (Figure 1a).

At DOL 28, no differences were reported among neonates born to symptomatic vs asymptomatic mothers regarding SARS-CoV-2 horizontal transmission, regardless of indoor environments and protective equipment used at home (Table 4). In the groups of the symptomatic mothers, we found a higher rate of use of disinfectant for baby bottle sterilization (Table 4). At DOL 28, 23.5% of the neonates was breastfed exclusively, 32.4% received infant formula, and 44.1% both maternal milk and infant formula (Figure 1b).

## 4. Discussion

In this study, we described the effects of the separation, immediately after birth, of the mother-infant dyad in case of maternal SARS-CoV-2 infection. We recorded no cases of neonatal infection due to horizontal transmission and one case of vertical transmission. On the other hand, the early separation of the dyad negatively affected the percentage of women who were able to breastfeed before hospital discharge and up to DOL 28. We show that a high adherence to recommended safety measures after discharge is associated with the absence of cases of SARS-CoV-2 horizontal transmission up to DOL 28.

The percentage of preterm births in our population of women positive for SARS-CoV-2 at delivery is high compared to the national rate [14]. This result is in line with data recently reported by the ItOSS [15] and suggests that pregnant women affected by SARS-CoV-2 should be subjected to a closer monitoring to prevent complication of premature birth. In addition, we have observed a higher percentage of pregnancy-related complications among the symptomatic mothers compared to the asymptomatic ones. These differences, although indicative, are not statistically significant, probably due to the small number of patients included in the study. In particular, although not significant, we found that symptomatic mothers were more likely to experience premature delivery compared to the asymptomatic ones, this result being in line with those of the current literature [16]. This data might be interpreted considering a general worsening of maternal clinical conditions which might have led to premature delivery [16].

We also observed a significantly higher percentage of symptomatic mothers among the ones with a higher educational level, probably reflecting more social contacts. However, our center is the referring obstetrical unit for women with SARS-Cov2 infection of a specific geographical area, thus limiting the generalizability of the observed finding. Of note, sociodemographic characteristics have been shown to have an impact on perinatal SARS-CoV-2 infection outcomes also in the neonatal population [17]. Our results showed that early and temporary separation of the dyad is not associated with horizontal transmission of SARS-CoV-2 from the mothers to their newborns. Neonatal SARS-CoV-2 infection seems more commonly acquired postnatally, through environmental exposure [3]. The postnatal mother-to-infant transmission of SARS-CoV-2 has been investigated by a few noncontrolled studies [3,4]. A metanalysis on 176 cases of confirmed neonatal SARS-CoV-2 infection reported that most neonatal infections were due to environmental/horizontal transmission and that mother-neonate rooming-in (i.e., the lack of mother-neonate separation soon after birth) was associated with a higher incidence of SARS-CoV-2 infection occurring after the first 72 h of life [3]. A large retrospective cohort analysis conducted in New York City reported two (2%) cases of neonatal infection when neonates roomed-in with their mothers [18]. On the other hand, a prospective cohort multicenter study on the risk of postnatal transmission of SARS-CoV-2 conducted in North Italy concluded that rooming-in practice, if protected (handwashing before caring for the baby, surgical mask, baby’s bed 2 m away from the mother), does not significantly impact viral transmission and should actually be encouraged [11]. In this study, no cases of neonatal infection were recorded in the subgroup of asymptomatic or mildly symptomatic mothers, but one newborn contracted the infection during rooming-in with the subgroup of symptomatic mothers.

The early separation of the dyad adopted in our management negatively affected the percentage of women who were able to breastfeed, with only a mild recovery in total breastfeeding rate being observed after the mother-infant reunification probably due to the decrease in breastfeeding support that occurred during the pandemic [19]. Among our population, we reported a very low percentage of newborns receiving exclusively maternal milk (2.7%) at discharge; during the same period in which this study was conducted, the percentage of women who breastfed at discharge after delivering a full-term baby with no pathological reports nor SARS-CoV-2 infection was about 60%, in line with central Italy’s previously published data [20]. At one month follow-up, we reported an increase of the percentage of neonates receiving exclusive breast milk (23.5%), despite being below the rates reported in the literature [20]. When we first designed the study, we did not include a control group; thus, data on feeding methods at one month after discharge in a similar population of full-term neonates born in our hospital are not available. The WHO recommends breastfeeding of infants and young children also in case of suspected or confirmed maternal SARS-CoV-2 infection [21]. The beneficial properties of breastfeeding including the practice of skin-to-skin care and the transfer of protective maternal antibodies via breast milk (especially secretory IgA (sIgA) and, to a lesser extent, IgM and IgG isotype immunoglobulins) are well established [22]. Recent evidence indicates that breastfeeding does not seem to be associated with neonatal SARS-CoV-2 infection because viral transmission through the milk, if any, should be rare [23] and because a robust sIgA-dominant SARS-CoV-2 antibody response is detectable in human milk soon after infection in a significant majority of individuals [24], suggesting a possible protection granted by the mothers to their infants [25,26,27,28,29,30]. Moreover, there is evidence in vitro suggesting whey proteins in human breastmilk as a direct-acting inhibitor of SARS-CoV-2 infection and replication [31]. While it was demonstrated that a healthy mother-infant dyadic process positively impacts the infants’ behavioral, cognitive, and socioemotional development [32], the possible long term effects of neonatal SARS-CoV-2 early infection, including neurological implications, are obviously unknown [33]. Further studies are advocated to establish if maternal perinatal infection confers immunological protection to the newborn and to evaluate possible maternal and neonatal long-term effects.

To our knowledge, this is the first study analyzing the short and long-term consequences of early and transient separation of the maternal-infant dyad. To better define the actual impact of separation or nonseparation of the dyad on the risk of neonatal infection, a double-blind randomized clinical trial would be needed, although this is not feasible for ethical reasons. Therefore, we believe that our data can contribute to the knowledge on this topic, serving as a reference group. However, if our findings suggested that the separation of the dyad is not associated with significant short-term advantages in a high-income country, it might be even harmful in low-income and middle-income countries [34].

Our results should be interpreted considering several limitations. Despite the limited number of cases described and the monocentric study design, Policlinico Umberto I Hospital is the reference birth center for COVID-19-positive pregnant women serving a large area of the Lazio region in Italy, and it well reflects the situation of the territory. In our study, data collection covered the neonatal period. Finally, data on placentas, amniotic fluid and their pathological reports among mothers with SARS-CoV-2 during pregnancy were not available. Studying histologic changes in the placenta of mothers with SARS-CoV-2 could be helpful to provide more of an understanding about this infection’s impact on pregnancy and vertical transmission, while the detection of the virus or specific antibodies in other neonatal biological samples (including blood, saliva, rectal swab) would be helpful to better define neonatal infection. Finally, we do not provide information about the presence of the virus in maternal milk of the enrolled mothers, although the current literature agrees on the safety of breastfeeding because the virus in the milk, if present, is not able to replicate and infect the neonate [35,36].

## 5. Conclusions

In a setting of early separation of the dyad, no cases of postnatal infection were observed. This policy is associated with a low rate of children receiving breast milk during the neonatal period. Although we do not know the long-term effects of SARS-CoV-2 neonatal infection, we are aware of the short- and long-term beneficial effects of breast milk; therefore, we believe that it is essential to encourage breastfeeding, even in case of mothers with COVID-19, when all safety measures are ensured.

## Figures and Tables

**Table 1:** Demographics and clinical characteristics of mothers with confirmed infection by SARS-CoV-2 at the delivery.

	All Mothers SARS-CoV-2 + (*n* = 37)	Asymptomatic Mothers (*n* = 10)	Symptomatic Mothers (*n* = 27)
Age, median years (min-max range)	31 (17–45)	33 (23–38)	31 (17–45)
Ethnicity			
Caucasian	25 (67.6)	4 (40.0)	21 (77.8)
Asian	8 (21.6)	4 (40.0)	4 (14.8)
African	1 (2.7)	1 (10.0)	0 (0)
Other	3 (8.1)	1 (10.0)	2 (7.4)
Smoke during pregnancy	2 (5.4)	0 (0)	2 (7.4)
Educational level ^a^	21 (56.8)	2 (20.0) *	19 (70.4)
Blood type			
Zero positive	16 (43.2)	4 (40.0)	12 (44.4)
Zero negative	4 (10.8)	1 (10.0)	3 (11.1)
A positive	15 (40.5)	4 (40.0)	11 (40.7)
B positive	2 (5.4)	1 (10.0)	1 (3.5)
Pregnancy-releated complications ^b^	14 (37.8)	2 (20.0)	12 (44.4)
Gestational diabetes	7 (18.9)	2 (20.0)	5 (18.5)
Thyroid dysfuncion	3 (8.1)	1 (10.0)	2 (7.4)
Pregnancy-induced hypertension	2 (5.4)	0 (0)	2 (7.4)
Urogenital tract infections	4 (10.8)	1 (10.0)	3 (11.1)
Premature birth	4 (10.8)	0 (0)	4 (14.8)
Intrapartum antibiotics	6 (16.2)	2 (20.0)	4 (14.8)
Intrauterine growth restriction	2 (5.4)	1 (10.0)	1 (3.7)
Antenatal steroids ^c^	4 (10.8)	0 (0)	4 (14.8)
Cesarean section	18 (48.6)	3 (30.0)	15 (55.6)

**Notes.** (a) At least secondary school; (b) At least one among: gestational diabetes, thyroid dysfunction, pregnancy-induced hypertension, urogenital tract infections, premature birth, intrapartum antibiotics, intrauterine growth restriction; (c) Intramuscular steroid cycle in one or two doses of 12 mg over a 24 h period; * vs. Symptomatic mothers *p value* < 0.05. Data were expressed as number (%), when not specified.

**Table 2:** Clinical features of symptomatic mothers with confirmed infection by SARS-CoV-2 at the delivery.

N. 27	
Fever	14 (51.9)
Anosmia	12 (44.4)
Ageusia	7 (25.9)
Myalgias	7 (25.9)
Vertigo	1 (3.7)
Cough	7 (25.9)
Dyspnea	3 (11.1)
Diarrhea	2 (7.4)
Vomit	1 (3.7)
Pharyngodynia	2 (7.4)
Cold/Sinusitis	3 (11.1)
Headache	1 (3.7)
Systemic Inflammatory Syndrome	1 (3.7)

**Notes.** Data were expressed as number (%).

**Table 3:** Characteristics of newborns from mothers with confirmed SARS-CoV-2 infection at the delivery.

	All Newborns (*n* = 37)	Neonate Born to Asymptomatic Mothers (*n* = 10)	Neonate Born to Symptomatic Mothers (*n* = 27)
Gestational age, median weeks (min-max range)	39 (32–42)	39 (38–41)	39 (32.42)
Birth weight, median grams (min-max range)	3235 (1880–4075)	3178 (2605–3830)	3240 (1880–4075)
Small for gestational age	5 (13.5)	1 (10.0)	4 (14.8)
Male gender	25 (67.6)	2 (20.0)	10 (37.0)
Fetal distress ^#^	2 (5.4)	0 (0)	2 (7.4)
pH on cord blood, median (min-max range)	7.3 (7.0–7.5)	7.3 (7.2–7.4)	7.3 (7.0–7.5)
5′ Apgar score, median (min-max range)	10 (7–10)	10 (9–10)	10 (7–10)
Body birth weight gain, median days (min-max range)	10 (0–17)	13 (11–17)	10 (0–16)
Blood Type			
Zero positive	14 (37.8)	4 (40.0)	10 (37.0)
Zero negative	4 (10.8)	0 (0)	4 (14.8)
AB positive	1 (2.7)	0 (0)	1 (3.7)
A positive	14 (37.8)	4 (40.0)	10 (37.0)
B positive	4 (10.8)	2 (20.0)	2 (7.4)
Length of hospital stays, median days (min-max range)	12 (4–55)	12 (5–31)	11 (4–55)

**Notes.** # cardiotocograph alteration and/or amniotic fluid “sludge”. Data were expressed as number (%), when not specified.

**Table 4:** Indoor environments and protective equipment used at home by caregivers of neonates born to SARS-CoV-2 positive mothers.

	All Mothers SARS-CoV-2 + (*n* = 33)	Asymptomatic Mothers (*n* = 8)	Symptomatic Mothers (*n* = 25)
At least one cohabitant positive for SARS-CoV-2	22 (66.7)	5 (62.5)	17 (68.0)
At least one cohabitant symptomatic for SARS-CoV-2	15 (45.5)	2 (25.0)	13 (52.0)
Metropolitan residence	26 (78.8)	7 (87.5)	19 (76.0)
Number of cohabitants, median (min-max range)	3 (1–7)	3 (2–5)	3 (1–7)
Number of bathrooms, median (min-max range)	1 (1–2)	1 (1–1)	1 (1–2)
External caregivers (not-cohabitants) negative for SARS-CoV-2	2 (6.1)	1 (12.5)	1 (4.0)
Use of surgical mask during baby care	28 (84.8)	7 (87.5)	21 (84.0)
Use of N95 mask during baby care	24 (72.7)	6 (75.0)	18 (72.0)
Use of glow during baby care	18 (54.5)	4 (50.0)	14 (56.0)
No use of PPE during baby care	5 (15.2)	1 (12.5)	4 (16.0)
Use of at least one PPE during baby care	28 (84.8)	7 (87.5)	21 (84.0)
Use at the same time of two PPE during baby care	25 (75.8)	6 (75.0)	19 (76.0)
Simultaneous use of three PPE during baby care	17 (51.5)	4 (50.0)	13 (52.0)
Hand washing before and after baby care	33 (100)	8 (100)	25 (100)
Cleaning of surfaces in contact with the newborn	30 (90.9)	6 (75.0)	24 (96.0)
Method for baby bottle sterilization			
Steam	21 (63.6)	3 (37.5)	18 (75.0)
Disinfectant	9 (27.3)	0 (0.0) *	9 (37.5)

**Notes.** PPE (Personal Protective Equipment: surgical mask, N95 mask and glow). * vs. Symptomatic mothers *p* value < 0.05. Data were expressed as number (%), when not specified.

## Data Availability

Data are available upon reasonable request. All data relevant to the study are included in the article. Access to raw data would be provided upon request.
